# Impact of protocolized diuresis for de-resuscitation in the intensive care unit

**DOI:** 10.1186/s13054-020-2795-9

**Published:** 2020-02-28

**Authors:** Brittany D. Bissell, Melanie E. Laine, Melissa L. Thompson Bastin, Alexander H. Flannery, Andrew Kelly, Jeremy Riser, Javier A. Neyra, Jordan Potter, Peter E. Morris

**Affiliations:** 1 Department of Pharmacy Services, Neuro-Pulmonary Division, University of Kentucky, 800 Rose Street, H110, Lexington, KY 40536 USA; 2 College of Pharmacy, University of Kentucky, 800 Rose Street, H110, Lexington, KY 40536 USA; 3 College of Medicine, Department of Internal Medicine, Division of Pulmonary, Critical Care and Sleep Medicine, University of Kentucky, 740 S. Limestone, Lexington, KY 40536 USA; 4 Performance Analytics Center of Excellence, University of Kentucky, 800 Rose Street, H110, Lexington, KY 40536 USA; 5 College of Medicine, Department of Internal Medicine, Bone and Mineral Metabolism, University of Kentucky, 800 Rose Street, MN668, Lexington, KY 40536 USA; 6 Department of Pharmacy Services, Beaumont Hospital, 3601 W 13 Mile Road, Royal Oak, MI 48073 USA

**Keywords:** Critical illness, Diuretics, Resuscitation, Fluid therapy, Pharmacists, Mechanical ventilation

## Abstract

**Objective:**

Administration of diuretics has been shown to assist fluid management and improve clinical outcomes in the critically ill post-shock resolution. Current guidelines have not yet included standardization or guidance for diuretic-based de-resuscitation in critically ill patients. This study aimed to evaluate the impact of a multi-disciplinary protocol for diuresis-guided de-resuscitation in the critically ill.

**Methods:**

This was a pre-post single-center pilot study within the medical intensive care unit (ICU) of a large academic medical center. Adult patients admitted to the Medical ICU receiving mechanical ventilation with either (1) clinical signs of volume overload via chest radiography or physical exam or (2) any cumulative fluid balance ≥ 0 mL since hospital admission were eligible for inclusion. Patients received diuresis per clinician discretion for a 2-year period (historical control) followed by a diuresis protocol for 1 year (intervention). Patients within the intervention group were matched in a 1:3 ratio with those from the historical cohort who met the study inclusion and exclusion criteria.

**Results:**

A total of 364 patients were included, 91 in the protocol group and 273 receiving standard care. Protocolized diuresis was associated with a significant decrease in 72-h post-shock cumulative fluid balance [median, IQR − 2257 (− 5676–920) mL vs 265 (− 2283–3025) mL; *p* < 0.0001]. In-hospital mortality in the intervention group was lower compared to the historical group (5.5% vs 16.1%; *p* = 0.008) and higher ICU-free days (*p* = 0.03). However, no statistically significant difference was found in ventilator-free days, and increased rates of hypernatremia and hypokalemia were demonstrated.

**Conclusions:**

This study showed that a protocol for diuresis for de-resuscitation can significantly improve 72-h post-shock fluid balance with potential benefit on clinical outcomes.

## Introduction

Early intravenous (IV) fluid resuscitation is a necessary tool to improve hemodynamic stability and organ perfusion and possibly decrease mortality in critically ill patients admitted to the intensive care unit (ICU) [1, 2]. However, the benefit of continued fluid administration after the first 24–48 h is unclear. Paradoxically, a positive fluid balance secondary to excess fluid accumulation has been associated with diverse and persistent detriment on a multitude of organ systems [3]. Perpetuating clinical harm has been demonstrated on pulmonary and renal function, as well as important clinical outcomes such as mortality and length of stay [1]. Despite the growing body of evidence supporting the adverse aspects of positive fluid balance, fluid overload remains common in ICU patients [4].

One approach to correcting fluid balance is shifting focus onto the post- or de-resuscitation period with appropriate diuresis, or renal replacement therapy (RRT) in those non-responsive to diuresis, once hemodynamic stability is achieved [5]. Effective diuresis may be challenged by many hindrances. An overall lack of standardization exists in identification of fluid-overloaded patients as optimal transition times between fluid resuscitation and fluid removal are not clear, and clinical signs of fluid overload are delayed relative to onset of organ damage [5–7].

Standard of care diuretic treatment regimens may be inadequate via sustained delays in initiation from shock resolution or inadequate dosing and follow-up. Additionally, apprehension for side effects can be seen, including serum creatinine rises and new onset acute kidney injury (AKI). However, the preponderance of adverse event data surrounding these medications is found in non-critical care populations, frequently non-translatable to patients in the ICU [8].

Previous protocols guiding volume removal in the critically ill can be found in specific populations including acute decompensated heart failure, AKI, or RRT weaning, with protocolized approaches often improving clinical outcomes versus standard of care [9–11]. Further, while limited evidence is available steering diuretic de-resuscitation in the broad ICU population, protocols have relied upon dated monitoring parameters, including central venous or pulmonary artery occlusion pressures [12–14]. In this study, we aimed to evaluate the impact of a novel diuresis protocol utilizing common bedside monitoring parameters and simplified loop diuretic dosing on cumulative fluid balance over the first 72 h following hemodynamic stability, as compared to standard of care.

## Materials and methods

### Patient selection

This was a pilot study to evaluate a service line level change in diuresis practice. Patients requiring mechanical ventilation with a net-positive or -even cumulative fluid or clinical signs of fluid overload determined via chest X-ray or physical exam between April 1, 2018, and April 1, 2019, received the diuresis protocol (see Additional file 1). Inclusion and exclusion criteria are summarized in Additional file 1. Patients were assessed for inclusion and exclusion daily while in the ICU. In order to approximate an experimental design using observational electronic health record (EHR) data, each patient visit within the intervention group was matched to three patient visits meeting the above inclusion and exclusion criteria from the historical time period of all Medical ICU admits between January 2016 and December 2017 who received furosemide. Diuresis practices in the historical group were non-protocolized and left to physician discretion. Patients who met the inclusion criteria from the historical cohort who were not matched with a patient from the intervention group were excluded from the analysis to prevent significant heterogeneity between groups.

### Study intervention

Patient identification occurred by the clinical pharmacist 7 days per week in collaboration with the medical team. After identification of appropriate patients for inclusion a net 24 h fluid balance (ranging from − 1000 mL to − 3000 mL) was established during interdisciplinary rounds which was divided into three shift goal fluid balance targets to assess at 8-h intervals. Upon establishment of goal, diuretic orders were entered, with dose selection based on previous diuretic exposure and baseline renal function. Orders included conditional diuretic orders if shift fluid balance targets were not met, basic metabolic panels, goal parameters, and hold parameters for adverse events (see Additional file 1). Combination diuresis was permitted once the maximum dose of furosemide was reached (200 mg IV) or potential hypernatremia. Available options included metolazone 10 mg oral or chlorothiazide 500 mg IV in instances when no enteral access was available. Indications for continuous infusion diuresis included a lack of response to 200 mg or failure of sustained diuretic response resulting in failure to achieve goal fluid balance.

In order to ensure appropriate compliance during overnight hours with decreased staffing ratios, an order set was created requiring nursing evaluation of urine output at the designated intervals. Conditional medication orders could be activated by the bedside nurse based on individual patient response and pharmacist-driven goal parameters. Diuresis hold parameters were established to minimize adverse events. The overall management of patients outside of diuresis protocol was left to physician discretion.

Given the paucity of evidence surrounding diuresis in this population, investigators involved in this study performed an interim analysis to promote a quality improvement corollary to the protocol. A data monitoring committee (DMC) was formed for data analysis after 50% of chronologic study completion. The DMC consisted of the division chief, independent statistical committee (ISC), and non-committee physicians, pharmacists, and nursing. Approximately 6 months from protocol initiation, the ISC performed data extraction which was brought forward to the DMC, without statistical analysis. A protocol modification occurred per the request of the DMC (see Additional file 1). This study protocol and modification were approved by the institutional review board. As this project was considered a quality improvement initiative, a waiver of informed consent was granted.

### Study outcomes

The primary outcome of this study was the net cumulative fluid balance 72 h following shock resolution. Secondary outcomes included ICU mortality, ICU length of stay, hospital length of stay, ventilator-free days, incidence of AKI (defined by KDIGO criteria), and the incidence of a severe metabolic disturbance including hypokalemia, hypernatremia, or de novo metabolic alkalosis, defined as a potassium < 3 mmol/L, sodium > 150 mmol/L, or bicarbonate > 40 mmol/L with a pH of > 7.50, respectively. Ventilator-free days were defined as the number of days from day 1 to day 28 in which a patient was able to breathe without assistance with death as a competing risk with an assignment of zero free days. For time-dependent interventions, medication administration record medication scans were utilized for medication-related times, respiratory therapy documentation was utilized for ventilator therapy, while admission, transfer, and discharge orders were collected for durations of stay.

### Statistical analysis

From our previous study of diluent change in the medical ICU, the average fluid balance in our patients at 72 h was positive 2.4 ± 5.1 l [15]. Based on these data, we calculated a sample size of 104 patients in each group to achieve a ≥ 2-l decrease in fluid balance at 72 h post-shock, maintaining an 80% power and an alpha of 0.05.

Continuous data were assessed for distribution and evaluated via *t* test or Mann-Whitney *U*, as appropriate. Chi-square or Fisher’s exact were utilized for categorical data. Data for analysis was pulled by a data analyst and validated with prospectively collected data, with discrepancies resolved by the analyst. The same inclusion and exclusion criteria used to enroll patients in the protocol were applied to selection of the control patients in the pre-protocol group. Mahalanobis distance matching was used to measure similarities of each patient in the control and protocol group. Age, gender, insurance type, home county classification, admission source, diagnosis-related group (DRG) weight, sequential organ failure assessment (SOFA) score at time of diuresis initiation, baseline serum creatinine prior to first dose of furosemide, pre-diuretic fluid balance, time from ventilator to first diuretic administration, pre-diuretic vasopressor administration, chronic obstructive pulmonary disease (COPD) diagnosis, and acute respiratory distress syndrome (ARDS) diagnosis were used as matching variables in the distance calculation. Nearest neighbor matching was then used to select the three control visits “closest” to each protocol visit, based on the distance calculation. The utilization of DRG was chosen by data analysis experts to bolster the validity of the severity of illness scores between groups. Further, a test of interaction was performed for patient enrollment pre- and post-protocol modification regarding the magnitude of difference on 72 h fluid balance.

A logistic regression model was defined a priori to be built for all-cause mortality. Forward selection was utilized with variables included in the model if *p* < 0.05 in the univariate analysis or if deemed biologically plausible and clinically relevant. These initial variables incorporated into the model included SOFA score, DRG weight, age, intervention versus standard therapy assignment, mechanical ventilation time to initiation of first dose of furosemide, net cumulative fluid balance prior to furosemide, and vasoactive therapy. If the intervention group was not to be identified as a significant covariate, it was predetermined that such would be manually entered into the final model to ascertain the point estimate. Collinearity was assessed with the use of variance inflation factors while goodness of fit was assessed with the Hosmer-Lemeshow test.

Given the potential for pertinent changes in clinical practice that are unrelated to the protocol, an interrupted time series was performed. Further, given the subjective nature of the inclusion criterion clinical signs of fluid overload determined via chest X-ray or physical exam, a subgroup analysis was performed including only those included based on objective volume status (net positive cumulative fluid balance at furosemide start). A subgroup was also collected for pre- and post-protocol amendments to assure no significant impact on clinical outcome.

## Results

**Fig. 1:**
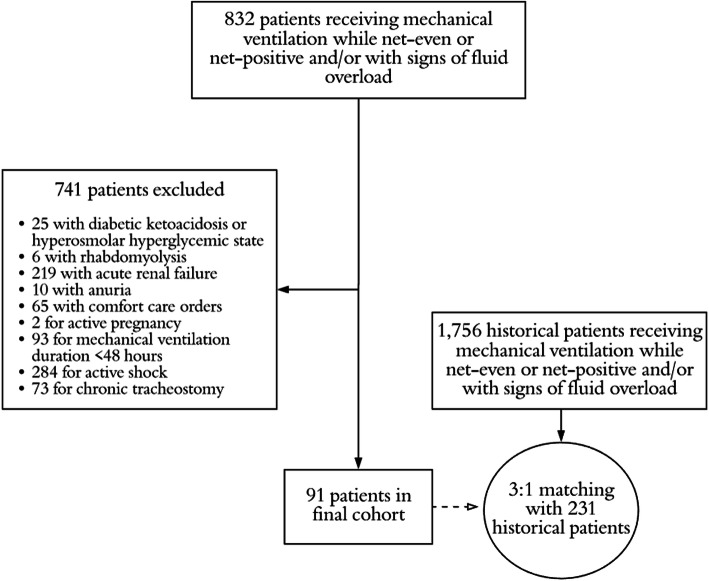
Selection of patients for study population

**Fig. 2:**
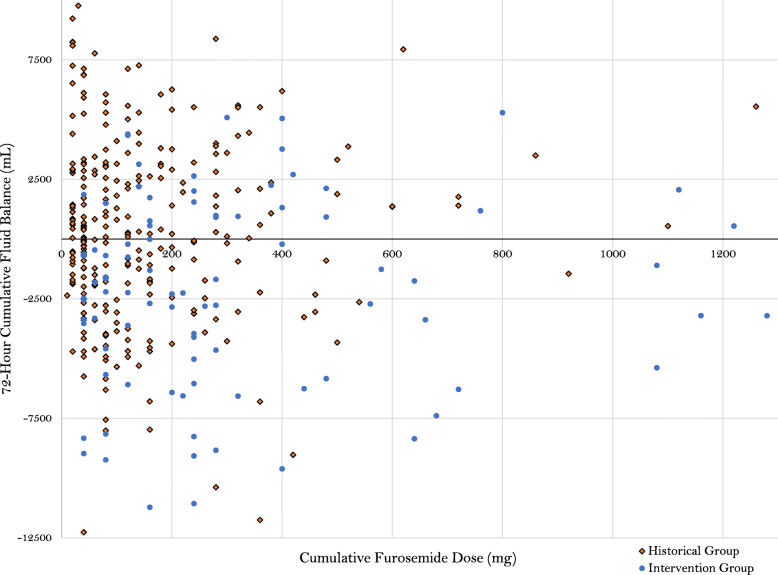
Furosemide dose and 72-h cumulative fluid balance per group

Over the study period, 832 patients met criteria for inclusion upon screening, of which, 741 were excluded based on pre-defined exclusion criteria (Fig. 1). A total of 273 standard therapy patients who met study criteria were matched 3:1 to patients in the intervention group (*n* = 91), for a total of 364 study patients. The matching procedures resulted in balanced groups, based on the pre-defined variables used in the matching algorithm (Table 1). Further, no major difference in other baseline clinical criteria was found with the exception of a higher arterial pH in the intervention group, as well as a higher incidence of rhabdomyolysis on admission (see Additional file 1). No difference was demonstrated in the utilization of concomitant medications, other than a higher incidence of use of intravenous anti-viral medications in the protocol group (Table 2). Regarding diuretic exposure, the diuresis protocol group received a higher dose of furosemide upon initiation, day 1–3, and cumulatively; however, diuretic dosing and patient response was variable (Fig. 2). More patients in the protocol group received concomitant metolazone or acetazolamide therapy, while the standard therapy group had more adjunctive albumin use.

**Table 1:** Baseline characteristics

Parameter	Historical cohort (*n* = 273)	Intervention cohort (*n* = 91)	*p* value
Matching parameter demographics			
Age (years)^*a*^	58 (48–68)	58 (46–70)	0.711
Male gender^*b*^	134 (49.1)	49 (53.8)	0.431
Medicare payer^*c*^	134 (49.1)	43 (47.3)	0.935
Medicaid payer^*c*^	97 (35.5)	36 (39.5)	
Commercial payer^*c*^	32 (11.7)	10 (10.9)	
Self-pay or government payer^*c*^	9 (3.3)	2 (2.2)	
Rural county^*b*^	33 (12.1)	9 (9.8)	0.262
Urban area^*b*^	105 (38.5)	28 (30.8)	
Urban cluster^*b*^	135 (49.5)	54 (59.3)	
Non-matching parameter demographics			
Chronic kidney disease^*b*^	41 (15.0)	11 (12.1)	0.489
Cirrhosis^*b*^	40 (14.7)	8 (8.8)	0.152
Matching critical illness parameters and comorbidities			
Cumulative fluid balance at furosemide start (mL)^*a*^	2243 (0–5381)	1411 (− 124–4438)	0.161
Vasopressor utilization prior to furosemide^*b*,*d*^	119 (43.6)	49 (53.8)	0.89
Time MV prior to furosemide (hours)^*a*^	45.5 (22–83)	52 (30.5–104)	0.155
Diagnostic-related group weight^*a*^	5.1 (2.3–5.9)	5.6 (2.4–6.3)	0.167
Prior SCr to furosemide (mg/dL)^*a*^	0.96 (0.74–1.29)	0.95 (0.75–1.44)	0.598
Sequential Organ Failure Assessment score ^*a*^	6 (4–8)	6 (4–8)	0.875
Chronic obstructive pulmonary disease^*b*^	64 (23.4)	25 (27.5)	0.439
Acute respiratory distress syndrome^*c*^	16 (5.9)	3 (3.3)	0.425
From emergency department (ED)^*c*^	65 (23.8)	14 (15.4)	0.301
From outside hospital^*c*^	96 (35.2)	39 (42.9)	
From outside hospital via ED^*c*^	62 (22.7)	25 (27.5)	
From other intensive care unit^*c*^	5 (1.8)	2 (2.2)	
From floor^*b*^	45 (16.5)	11 (12.1)	

*MV* mechanical ventilation

^*a*^Wilcoxon rank sum, median (interquartile range)

^*b*^Chi-square test; number (percentage)

^*c*^Fisher’s exact, number (percentage)

^*d*^Vasopressors including norepinephrine, epinephrine, or vasopressin

**Table 2:** Pharmacotherapy

Parameter	Historical cohort (*n* = 273)	Intervention cohort (*n* = 91)	*p* value
Furosemide dosing			
Starting dose (mg) ^*a*^	40 (20–40)	40 (40–40)	0.003
Day one total daily dose (mg) ^*a*^	40 (40–60)	80 (40–120)	< 0.0001
Day two total daily dose (mg)^*a*^	0 (0–40)	80 (20–120)	< 0.0001
Day three total daily dose (mg) ^*a*^	0 (0–20)	0 (0–80)	0.0007
Total cumulative dose (mg)^*a*^	80 (40–200)	240 (120–420)	< 0.0001
Conversion to continuous infusion ^*b*^	32 (11.7)	8 (8.8)	0.562
First to last dose furosemide (days) ^*a*^	4.9 (1.4–12.4)	4.8 (3.1–9.8)	0.165
Diuresis adjuncts			
Metolazone^*b*^	15 (5.5)	30 (32.9)	< 0.0001
Chlorothiazide^*c*^	48 (17.6)	6 (6.6)	0.402
Acetazolamide^*b*^	14 (5.1)	14 (15.4)	0.001
Albumin^*c*^	29 (10.6)	2 (2.2)	0.009
Day one potassium supplementation^*a*^	40 (40–60)	60 (40–80)	0.007
Day two potassium supplementation^*a*^	40 (40–60)	60 (40–100)	0.002
Day three potassium supplementation^*a*^	50 (40–80)	70 (60–100)	0.002
Other medication exposure			
Total nephrotoxin exposure^*a*^	1 (1–2)	1 (1–2)	0.288
Aminoglycoside^*b*^	27 (9.9)	8 (8.8)	0.758
Beta-lactam^*b*^	227 (83.2)	75 (92.4)	0.872
Intravenous antiviral^*b*^	11 (4.0)	12 (13.2)	0.002
ACE inhibitor and/or ARB^*b*^	49 (17.9)	13 (14.3)	0.421
Amphotericin B^*c*^	5 (1.8)	3 (3.3)	0.418
Intravenous sulfamethoxazole-trimethoprim^*c*^	19 (6.9)	4 (7.7)	0.465
Intravenous vancomycin^*b*^	153 (56.0)	51 (56.0)	1.000
Combination vancomycin and piperacillin-tazobactam^*b*^	88 (32.2)	30 (32.9)	0.897

*ACE* angiotensin-converting enzyme, *ARB* angiotensin receptor blocker

^*a*^Wilcoxon rank sum, median (interquartile range)

^*b*^Chi-square test; number (percentage)

^*c*^Fisher’s exact, number (percentage)

The median (IQR) fluid balance within this study at 72-h post-shock resolution was 265 mL (− 2283–3025) vs − 2257 mL (− 5676–920) in the historical and interventional cohorts, respectively (*p* < 0.0001) (Table 3). There was also a significant difference in 24- and 48-h fluid balance in the intervention group when compared to the historical cohort. The test of interaction demonstrated no statistical significance regarding those enrolled in the protocol before or after modification (see Additional file 1), and the subgroup analysis excluding those patients based on subjective clinical criteria (physical exam findings, concern for pulmonary edema) showed similar findings (see Additional file 1). In the interrupted time series accounting for potential practice variation over time, no significance was demonstrated relative to time before or after intervention (see Additional file 1). However, a significant difference was demonstrated in 72-h post-shock fluid balance with protocol use (see Additional file 1). For the secondary outcomes, while patients had an additional ventilator-free day in the intervention group, this difference was not statistically significant. Within the intervention cohort, there was a statistically significant increase in the rate of electrolyte disturbances, primarily driven by an increase in hypernatremia and hypokalemia, despite higher total potassium replacement in the intervention group.

**Table 3:** Clinical outcomes

Parameter	Historical cohort (*n* = 273)	Intervention cohort (*n* = 91)	*p* value
Clinical outcomes			
72 h fluid balance (mL)^*d*^	265 (− 2283–3025)	− 2257 (− 5676–920)	< 0.0001
48-h fluid balance (mL) ^*d*^	309 (− 1267–2434)	− 1799(− 3884–1092)	< 0.0001
24-h fluid balance (mL)^*a*^	101 (− 963–1622)	− 692 (− 1833–697)	0.0002
Ventilator-free days (days) ^*a*^	19 (10–22)	20 (15–23)	0.098
Overall adverse event^*b*,*e*^	74 (27.1)	37 (40.6)	0.015
Ventilator days (days) ^*a*^	8 (5–13)	5 (5–12)	0.441
Furosemide to extubation (hours) ^*a*^	70 (24–147)	58 (23–122)	0.282
Re-intubation rate^*b*^	57 (20.8)	17 (18.6)	0.652
ICU-free days (days) ^*a*^	17 (7–21)	19 (13–22)	0.030
ICU days (days) ^*a*^	8.6 (6.2–13.5)	8.1 (5.9–12.8)	0.513
In-hospital mortality^*c*^	44 (16.1)	5 (5.5)	0.008
Safety outcomes			
Bolus administration after furosemide^*c*^	4 (1.5)	0 (0)	0.576
Vasopressor administration after furosemide^*b*^	65 (23.8)	19 (20.9)	0.566
Tachyarrhythmia^*b*^	50 (18.3)	15 (16.4)	0.693
In-hospital mortality^*c*^	44 (16.1)	5 (5.5)	0.008
RRT receipt in ICU^*c*^	17 (6.2)	0 (0)	< 0.0001
RRT dependence at discharge^*c*^	14 (5.1)	0 (0)	0.025
Acute kidney injury^*f*^	62 (22.7)	22 (24.2)	0.775
Hypokalemia^*c*^	0	3 (3.3)	0.015
Hypernatremia^*b*^	19 (6.9)	19 (20.9)	0.001
Metabolic alkalosis^*c*^	3 (1.1)	1 (1.1)	1.000

^*a*^Wilcoxon rank sum, median (interquartile range)

^*b*^Chi-square test; number (percentage)

^*c*^Fisher’s exact, number (percentage)

^*d*^Student’s *t* test, average (standard deviation)

^*e*^Overall adverse event; serum creatinine rise, hypokalemia, hypernatremia, or metabolic alkalosis

^*f*^Acute kidney injury; serum creatinine 1.5 times baseline serum creatinine, serum creatinine increase of at least 0.3 mg/dL

In-hospital mortality in the intervention group was lower compared to the historical group (5.5% vs 16.1%; *p* = 0.008). There was also a higher rate of ICU-free days, with these patients having 2 more days free of ICU care (*p* = 0.03). In multivariable analysis, protocolized therapy was associated with a 75% (32–91%) decreased odds of hospital mortality after adjustment for SOFA, fluid balance upon furosemide initiation, time on mechanical ventilation prior to furosemide therapy, and age (see Additional file 1). Given known limitations of serum creatinine as a marker of kidney function during acute illness, a post hoc analysis was performed of RRT dependence at discharge. RRT dependence at discharge was found to be significantly higher in the standard therapy cohort compared to the protocol group.

Regarding protocol compliance, a total of 204 patient days on protocol were available for evaluation. The most common indication for a furosemide hold was due to protocol discontinuation (see Additional file 1). A total of 27 deviations occurred within the 204 patient days, 8 for a decrease in dosing frequency prior to protocol modification, 2 for doses administered despite hold criteria, 2 missed nursing activations of conditional orders, and 12 inappropriate holds, 7 of which for unknown reasons, 1 for nursing concern regarding furosemide interval, and 4 for urine output. Eighteen patient days required a dose adjustment per protocol, 11 of which were driven by conditional orders.

## Discussion

**Fig. 3:**
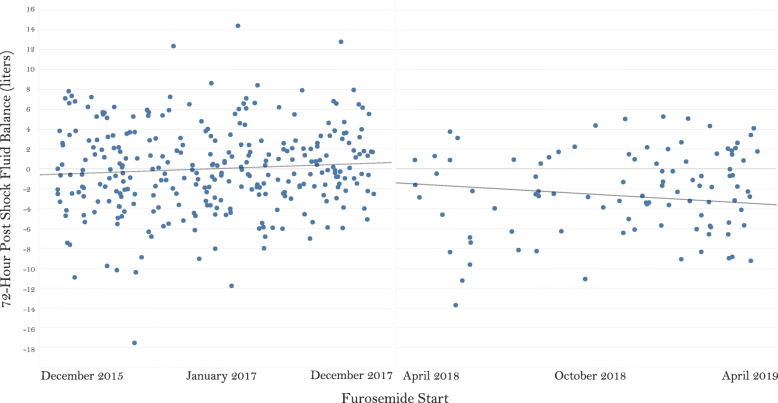
Interrupted time series analysis of 72-h post-shock fluid balance over time. Pre-intervention slope *R*^2^ = 0.0092, *p* = 0.099; post-intervention slope *R*^2^ = 0.018, *p* = 0.185

This study was the first to evaluate a volume de-resuscitation protocol utilizing pharmacologic diuresis in the medical intensive care unit. This study has several strengths, including the protocol with easily obtainable bedside monitoring parameters within the EHR, the multi-disciplinary approach to protocol development, utilization, and modification, frequency of monitoring, and selection of matching parameters. Several potential confounders on 72-h fluid balance were matched between groups, systematically decreasing between-group difference. Further, results of the interrupted time series showed no significant difference in slopes of fluid balance over time, while the association between improved 72-h post-shock fluid balance and intervention group remained significant (Fig. 3).

We demonstrated a significant decrease in 72 h cumulative fluid volume with the addition of a diuresis protocol in the critically ill. This correlates with previous protocols within acute respiratory distress syndrome and heart failure which demonstrated improved volume status with strategized diuresis without an increase in kidney failure [11, 14]. Unlike studies within the heart failure population, our protocol prioritized intermittent dosing to decrease intravenous access concerns and protocolized electrolyte and safety monitoring [14]. With such, a significant increase in the rate of hypernatremia and hypokalemia was seen within the intervention group. No statistically significant difference in duration of mechanical ventilation wean was found. This does not correlate with previous evidence within the critically ill population, demonstrating increased ventilator-free days with conservative volume management [14]. Comparatively, while our study utilized more specific titration strategies and common bedside monitoring parameters, this was a single-center, non-randomized study and likely underpowered to detect a difference in ventilator duration.

Key considerations to this study include a decrease in mortality and increased ICU-free days in the intervention group. Known correlates of mortality within the sepsis population, including baseline weight and admission source, were included as parameters within the regression model [16–18]. The variables previously correlated with mortality were accounted for in the matching criteria of this cohort. Studies demonstrate that almost ubiquitous organ dysfunction has been associated with positive volume status in the ICU. It is possible that the implication of volume de-resuscitation seen in the current study could be casually linked with mortality, in line with a vast number of previous studies demonstrating the impact of fluid status on survival rates aside of its effect on ventilator days; however, this study can only show correlation given the nature of its design. Particularly, patients in the intervention group also had a decrease in RRT dependence at discharge. RRT receipt prior to hospital discharge has been associated with progression to end stage renal disease, cardiovascular disease, and increased mortality [19, 20].

Regarding ventilator days, ventilation wean procedures are not standardized at this institution. Daily spontaneous breathing trials are performed in all patients who meet criteria; however, extubation orders are left to provider discretion. This lack of ventilator wean protocolization may have affected ventilator-free days between groups. However, reintubation rates were in alignment with previous studies with ranges 13.8–22.6% and were not significantly different between groups which supports relative uniformity on wean strategies [21].

Further of note, changes to the institutional nursing-driven electrolyte replacement protocol occurred mid-implementation (see Additional file 1). The protocol modification sought more aggressive potassium replacement; however, nursing adherence was not evaluated. As follow-up potassium evaluations were mandated with protocol implementation, it is possible that incidences of hypokalemia were increased secondary to more frequent monitoring relative to the historical cohort; however, frequency of serum potassium collections were not recorded. In regard to rates of hypernatremia, providers were permitted to request continuation of furosemide despite elevated sodium levels, likely resulting in the subsequent increased rate of metolazone use in the intervention group. There was a significant difference in cumulative fluid balance that was likely due to higher furosemide exposure in the intervention group, as demonstrated in previous protocols of furosemide in acute kidney injury [10]. The significant increase in episodes of hypernatremia and hypokalemia are predictable and reversible within this strategy. If replicated in future randomized trials, improvements in ICU length of stay and mortality may take precedence over concern for electrolyte abnormalities. Future protocol designs should account for these episodes of hypernatremia and hypokalemia with creation of more explicit electrolyte replacement rules. Further, electrolyte derangements may be of greater consideration in an alternative ICU population, including cardiothoracic/cardiology critical care. Patient-specific factors should be taken into consideration with implementation of this protocol.

A key limitation to this study is the lack of randomization and blinding. Given the nature of the protocol, blinding to the medical staff was not possible. A pre- and post-intervention study was chosen given the lack of blinding. It was anticipated that an overall change in practice may occur over the study timeframe given increased awareness of the detrimental effects of fluid overload and approach to diuretic dosing in critically ill patients, a phenomenon recently found in management of septic shock [22, 23]. However, given the limited time lapse between the historical group and protocol implementation and lack of emergence of guidelines regarding volume de-resuscitation, changes in overall approaches to care based on external factors were unlikely. To limit potential bias further, patients were matched on a large number of relevant variables and objective outcome measures were utilized, with the exception of the DRG weight. However, the authors opted for inclusion of this variable versus International Classification of Disease coding given its consideration for up to eight diagnoses, including the primary diagnosis, and up to six procedures performed during the stay, likely increasing its objectiveness versus retrospective chart review. Regardless, it is still possible for potential residual confounders on illness severity to have been missed. Given that volume overload and positive fluid balance may be markers of severity of illness rather than a parameter for early diuresis intervention, the differences in mortality and length of stay must be replicated in a larger, randomized controlled trial for confirmation. Worth nothing, true blinding in a randomized controlled trial would likely be unfeasible by nature of the protocol design and a parallel design could subject the trial to potential for a significant Hawthorne effect.

Protocol modifications in the study may also be seen as a potential limiting factor. However, in the subgroup analysis performed, protocol inclusion did not appear to significantly impact the primary result. Additionally, the inclusion rate appeared relatively low at 11%. Recent studies have demonstrated small recruitment rates within the critically ill [24, 25]. A significant portion of our patients were excluded for active vasoactive therapy or AKI. Clinical inertia is a consideration, particularly given this protocol’s pilot nature. Further, consideration must be made for a lag in adaptation, particularly in times of low staffing.

Lastly, the selection of outcome parameters is worth mentioning. We evaluated 72-h net cumulative fluid balance in accordance with previous literature; however, evidence suggests that fluid balance documentation is not always accurate. The utilization of EHR flowsheets decreases potential for error in ICU documentation. The frequency in documentation required via the protocol aligns with standard of care within the ICU. Recent studies have challenged the validity of net cumulative fluid balance in the ICU and its relationship to body weight or clinical signs of fluid overload [26, 27]. Because this practice is not tightly protocolized, we did not utilize body weight as a monitoring parameter. However, it is possible that daily weight monitoring would assist in clinical decision making and outcome measures.

This study demonstrated that a pharmacist-driven diuresis protocol of volume de-resuscitation was significantly associated with a lower cumulative fluid balance at 72 h post-shock. The addition of the diuresis protocol was likely effective for a multitude of reasons, including the overall increased awareness of avoidance of volume overload and tailored diuresis utilization, the standardization of doses and follow-up monitoring, as well as an increase in furosemide dosing as demonstrated in this study. However, with increased dosing of furosemide, increased rates of adverse events were found, namely hypernatremia and hypokalemia. Risk versus benefit of active volume de-resuscitation and electrolyte fluctuations must be considered. The increased mortality and decreased number of ICU-free days in the standard therapy group are hypothesis-generating, particularly given the lack of difference between-groups in ventilator-free days.

## Conclusion

Using a diuresis protocol for volume de-resuscitation, we demonstrated a significant decrease in net cumulative fluid balance at 72 h following shock resolution, with potential benefit on clinical outcomes including renal recovery, mortality, and ICU length of stay. Although this study supports the implementation of a diuresis protocol in the ICU, larger randomized controlled trials are needed to confirm or refute the potential benefits of de-resuscitation, through protocol-driven diuresis, on important patient centered outcomes, such as ICU length of stay, ventilator-free days, and in-hospital mortality, as suggested by observed associations in the present study.

## Supplementary information

**Additional file 1.** Supplementary Digital Content This file includes relevant study protocols, definitions, as well as subgroup analyses and additional informational tables beyond manuscript content.

## Data Availability

The datasets during and/or analyzed during the current study are available from the corresponding author on reasonable request.
