# Supplementary material for: Impact of protocolized diuresis for de-resuscitation in the intensive care unit
Source: Crit Care. 2020 Feb 28;24:70. doi: 10.1186/s13054-020-2795-9 (PMC7048112; doi:10.1186/s13054-020-2795-9)
Supplement: Supplementary file 1 — Additional file 1. Supplementary Digital Content This file includes relevant study protocols, definitions, as well as subgroup analyses and additional informational tables beyond manuscript content. [file 13054_2020_2795_MOESM1_ESM.pdf]

Supplementary Digital Content

Appendix A. Intensive Care Unit Electrolyte Replacement Protocol

Appendix B. Study Definitions

eFigure 1. Pre-Modification Diuresis Protocol

eFigure 2. Post-Modification Diuresis Protocol

eTable 1. Study Inclusion and Exclusion Criteria

eTable 2. Additional Baseline Characteristics

eTable 3. Primary Diagnosis Code Groups

eTable 4. Subgroup Analysis of Pre- and Post-Modification Enrollment

eTable 5. Subgroup Analysis for 72-Hour Post-Shock Fluid Balance

eTable 6. Interrupted Time Series Analysis

eTable 7. Logistic Regression for All-Cause Mortality

eTable 8. Indication for Furosemide Dose Holds in Intervention Group

Appendix A:

(a) Intensive Care Unit Electrolyte Replacement Protocol General Criteria

Summary

Standing electrolyte replacement protocols are available for use in adult patients admitted to an intensive care unit or progressive care upon direct physician order entry in the EMR.

Requirements

- Physician may order via physician order entry in EMR electrolyte replacement protocol for Potassium chloride, Magnesium sulfate, Phosphorous, or Calcium gluconate individually or in combination. This authorizes the nurse to order and administer all electrolyte replacement doses and laboratory orders as defined per protocol.
- Replacement protocols should NOT be used in patients with:
    a) serum creatinine (SCr) > 2 mg/dL
    b) urinary output < 30 mL/hr
    c) patients requiring hemodialysis
    d) weight < 50 kg
    e) patients undergoing targeted temperature management (therapeutic hypothermia)
- Physician should be notified if electrolyte deficiency does not correct after two rounds of electrolyte replacement in 24 hours.
- Nursing should preferentially use oral over intravenous replacement whenever possible.
    - Use oral replacement when the patient is getting other oral or per tube medications.
    - If the patient is actively receiving vasopressors, oral replacement can only occur after a discussion with the provider.

(b) Intensive Care Unit Electrolyte Replacement Protocol Potassium Chloride Replacement

- Telemetry monitoring is required for infusion rates > 10 mEq/hr.
- If serum potassium level < 3 mEq/L, order STAT magnesium level if no result in prior 24 hours.
- If both serum magnesium and potassium levels are low, initiate magnesium for 1 hour prior to starting potassium repletion.

**Serum Potassium Level (mEq/L)**	**Total Replacement**	**Central IV Administration** *(Rate 20 mEq/hr)*	**Peripheral IV Administration** *(Rate 10 mEq/hr)*	**Oral Administration** *ER Tablet*	**Oral Administration** *Nothing by mouth*	**Potassium Re-Check**
3.7 – 4	40 mEq	20 mEq/50 mL IV x 2 dose	10 mEq/100 mL IV every hour x 4 doses	10 mEq x 4 tablets PO	20 mEq x 2 packets PO/FT	No additional action
3.4 – 3.6	60 mEq	20 mEq/50 mL IV every hour x 3 doses	10 mEq/100 mL IV every hour x 6 doses	10 mEq x 6 tablets PO	20 mEq x 3 packets PO/FT	2 hours after replacement complete
3 – 3.3	80 mEq	20 mEq/50 mL IV every hour x 4 doses	10 mEq/100 mL IV every hour x 8 doses	10 mEq x 4 tablets PO every 4 hours x 2 doses	20 mEq x 2 packets PO/FT every 4 hours x 2 doses	2 hours after replacement complete
< 3 *STAT serum Mag if none in 24 hours*	100 mEq and notify physician	20 mEq/50 mL IV every hour x 5 doses	10 mEq/100 mL IV every hour x 10 doses	10 mEq x 4 tablets PO + 20 mEq IV every hour x 3 doses (central line only)[a]	20 mEq x 2 packets PO/FT + 20 mEq IV every hour x 3 doses (central line only)[b]	2 hours after replacement complete

a If peripheral IV access only: 10 mEq x 4 tablets PO plus 10 mEq/100 mL IV every hour x 6 doses; *b*If peripheral IV access only: 20 mEq x 2 packets PO/FT + 10 mEq/100 mL IV every hour x 6 doses; mEq, miliequivalents; mL, milliliters; IV, intravenous; PO, per mouth; FT, per feeding tube

(c) Intensive Care Unit Electrolyte Replacement Protocol Magnesium Sulfate IV Replacement

- If both serum magnesium and potassium levels are low, initiate magnesium repletion for 1 hour prior to starting potassium repletion.

**Serum Magnesium Level (mg/dL)**	**Total Magnesium Sulfate Replacement IV**	**Serum Magnesium Re-Check**[a,b]
1.5 – 1.8	2 grams/50 mL IV over 4 hour	No additional action
1.2 – 1.4	4 grams /100 mL IV x 2 each over 8 hours	2 hours after final infusion complete
< 1.2 (asymptomatic)	4 grams /100 mL IV x 3 each over 8 hours and notify physician	2 hours after final infusion complete
< 1.2 (symptomatic[a])	2 grams IVP, then 4 gm/100 mL IV x 3 each over 8 hours and notify physician	2 hours after final infusion complete

[a] If a scheduled lab draw which includes the electrolyte needing reassessment is within 4 hours after the total electrolyte replacement administration, no additional lab draw is needed. If no lab draw is scheduled, draw as instructed per protocol; [b] Symptoms of hypomagnesaemia include: 1) Neuromuscular - hyperexcitability (tremor, tetany, convulsions), weakness, apathy, delirium, and coma, 2) Cardiovascular - widening of the QRS and peaking of T waves with moderate magnesium depletion and atrial and ventricular arrhythmias with severe depletion, 3) Hypokalemia; mL, milliliters; IV, intravenous

(c) Intensive Care Unit Electrolyte Replacement Protocol Magnesium Oxide Oral Replacement

- If both serum magnesium and potassium levels are low, initiate magnesium repletion for 1 hour prior to starting potassium repletion.
- DO NOT replace magnesium orally unless the magnesium oxide is specifically ordered for the patient AND use caution if patient is having more than 3 loose bowel movements daily

**Serum Magnesium Level (mg/dL)**	**Total Magnesium Replacement PO (magnesium oxide)**	**Serum Magnesium Re-Check**[a,b]
1.5 – 1.8	400 mg PO/FT	No additional action
1.2 – 1.4	400 mg PO/FT every 4 hours x 2	2 hours after final infusion complete
< 1.2 (asymptomatic)	400 mg PO/FT every 4 hours x 3	2 hours after final infusion complete
< 1.2 (symptomatic[a])	Notify physician	2 hours after final infusion complete

[a] If a scheduled lab draw which includes the electrolyte needing reassessment is within 4 hours after the total electrolyte replacement administration, no additional lab draw is needed. If no lab draw is scheduled, draw as instructed per protocol; [b] Symptoms of hypomagnesaemia include: 1) Neuromuscular - hyperexcitability (tremor, tetany, convulsions), weakness, apathy, delirium, and coma, 2) Cardiovascular - widening of the QRS and peaking of T waves with moderate magnesium depletion and atrial and ventricular arrhythmias with severe depletion, 3) Hypokalemia; PO, by mouth; FT, per feeding tube

(d) Intensive Care Unit Electrolyte Replacement Protocol Phosphorous Replacement

- IV sodium phosphorus doses are rounded to the nearest 15 mmol. Available products will be:
- For administration via central line either 15mmol in 100 ml NS, 30 mmol in 100 ml NS or 45 mmol in 250 ml NS
- For administration via a peripheral line either 15 mmol in 250 ml NS, 30 mmol in 500 ml NS or 45 mmol in 500 ml NS

**Serum Phosphorous Level (mg/dL)**	**Sodium Phosphorus IV replacement**[a,b]	**Oral Administration** ***(Phospha 250 Neutral tablet, 8 mmol)***	**Oral Administration** ***Nothing by Mouth (PhosNaK packet, 8 mmol)***	**Serum Phosphate Re-Check** [c]
2.7 – 3	None	1 tablet PO two times daily x 2 doses	1 packet PO/FT two times daily x 2 doses	No additional action
2.1 – 2.6	0.16 mmol/kg IBW[e] (max 15 mmol) IV over 4 hours	1 tablet PO three times daily x 3 doses	1 packet PO/FT three times daily x 3 doses	No additional action
1 – 2	0.24 mmol/kg IBW[e] (max 30 mmol) IV x over 4 hours	2 tablets PO three times daily x 3 doses	2 packets PO/FT three times daily x 3 doses	2 hours after replacement complete
< 1	0.5 mmol/kg IBW[e] (max 45 mmol) IV over 6 hours and notify physician	0.16 mmol/kg IBW[e] (max 15 mmol)[d] IV over 4 hours + 2 tablets PO three times daily x 3 doses + notify physician	0.16 mmol/kg IBW[e] (max 15 mmol)[d] IV over 4 hours + 2 packets PO/FT three times daily x 3 doses + notify physician	2 hours after replacement complete

[a] IV phosphate repletion utilizes Sodium phosphate only. Potassium replacement should be done separately; [b]IV phosphorus dosing are based on IBW;[c]If a scheduled lab draw which includes the electrolyte needing reassessment is within 4 hours after the total electrolyte replacement administration, no additional lab draw is needed. If no lab draw is scheduled, draw as instructed per protocol; IBW, ideal body weight; IV, intravenous; mmol, millimole; kg, kilogram; PO, by mouth; FT, per feeding tube

(e) Intensive Care Unit Electrolyte Replacement Protocol Calcium Gluconate Replacement

**Ionized Calcium Level (mg/dL)**	**Total Calcium Replacement**[a]	**Serum Calcium Re-Check**[b,c]
3.4 – 4.6 (asymptomatic)	None	12 hours
3.4 – 4.6 (symptomatic[f])	Calcium gluconate 1 gm/100 mL IV over 30 min	2 hours after infusion completed
< 3.4	Calcium gluconate 2 gm/100 mL IV over 1 hour	2 hours after infusion completed

[a] Vesicant precautions: Central Line preferred but ok to give as peripheral infusions done separately; [b]Symptoms of acute hypocalcemia include: 1)Neuromuscular – tetany, paresthesias (peri-oral, extremities), muscle twitching, carpopedal spasms, seizures, laryngospasm, bronchospasm, 2) Cardiac - prolonged QT interval, hypotension, heart failure, arrhythmia, 3) Papilledema; [c]If a scheduled lab draw which includes the electrolyte needing reassessment is within 4 hours after the total electrolyte replacement administration, no additional lab draw is needed. If no lab draw is scheduled, draw as instructed per protocol

Appendix B:

I. Hypernatremia – serum sodium greater than 150 mmol/L
II. Net positive fluid balance - any volume status value above a 0 mL net volume
III. Nephrotoxins - aminoglycosides, amphotericin, beta-lactam therapy, intravenous antivirals, intravenous vancomycin, and sulfamethoxazole-trimethoprim.
IV. Shock resolution - freedom from vasopressor administration or bolus crystalloid administration for at least 12 hours.
V. Ventilator-free days - the number of days from day 1 to day 28 in which a patient was able to breathe without assistance with death as a competing risk with an assignment of zero free-days
VI. KDIGO AKI Criteria - serum creatinine 1.5 times serum creatinine prior to diuresis initiation, serum creatinine increase of at least 0.3 mg/dL within 48 hours, or urine output <0.5 mL/kg/hr for at least 6 hours

eFigure 1. Pre-Modification Diuresis Protocol

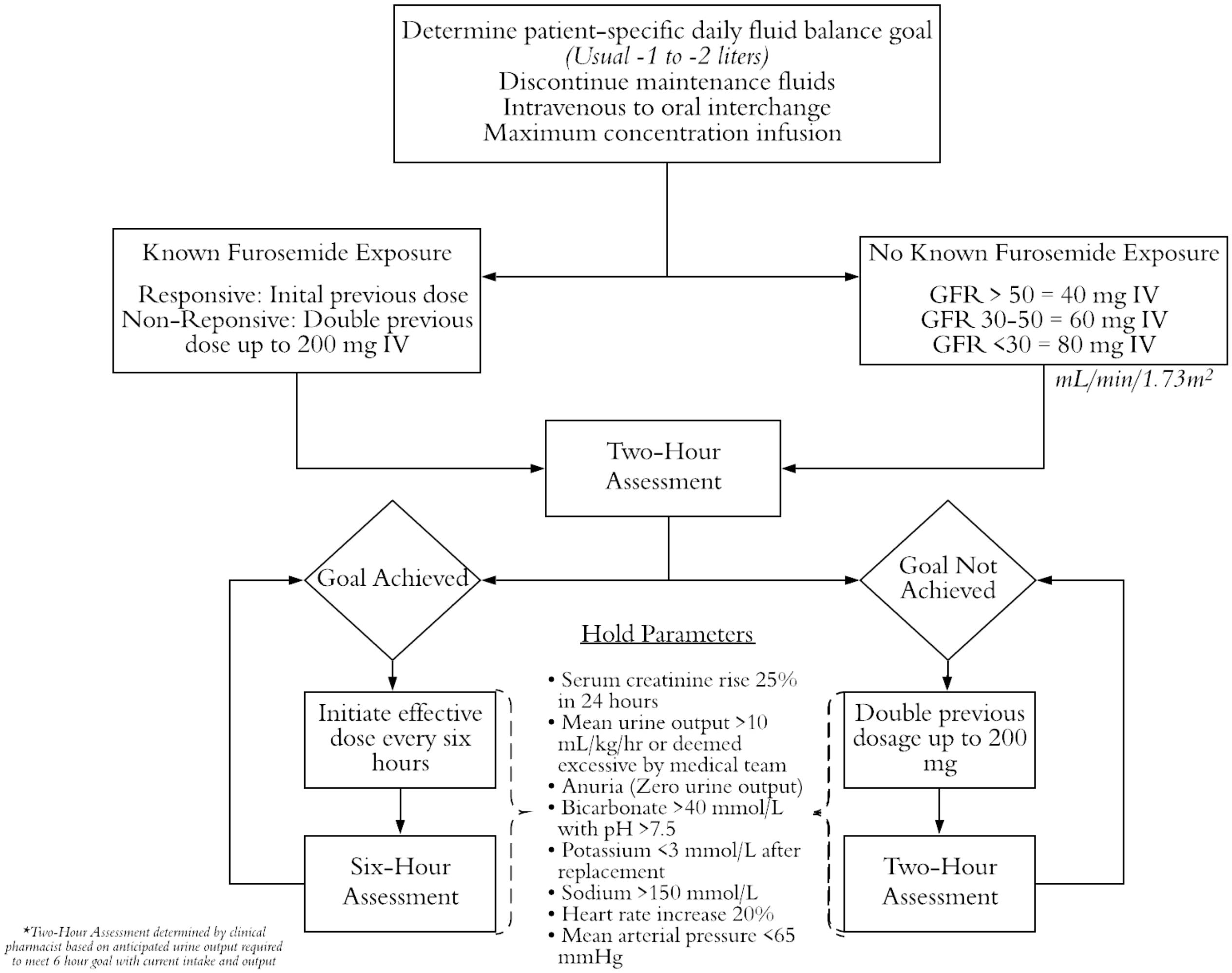

eFigure 2. Post-Modification Diuresis Protocol

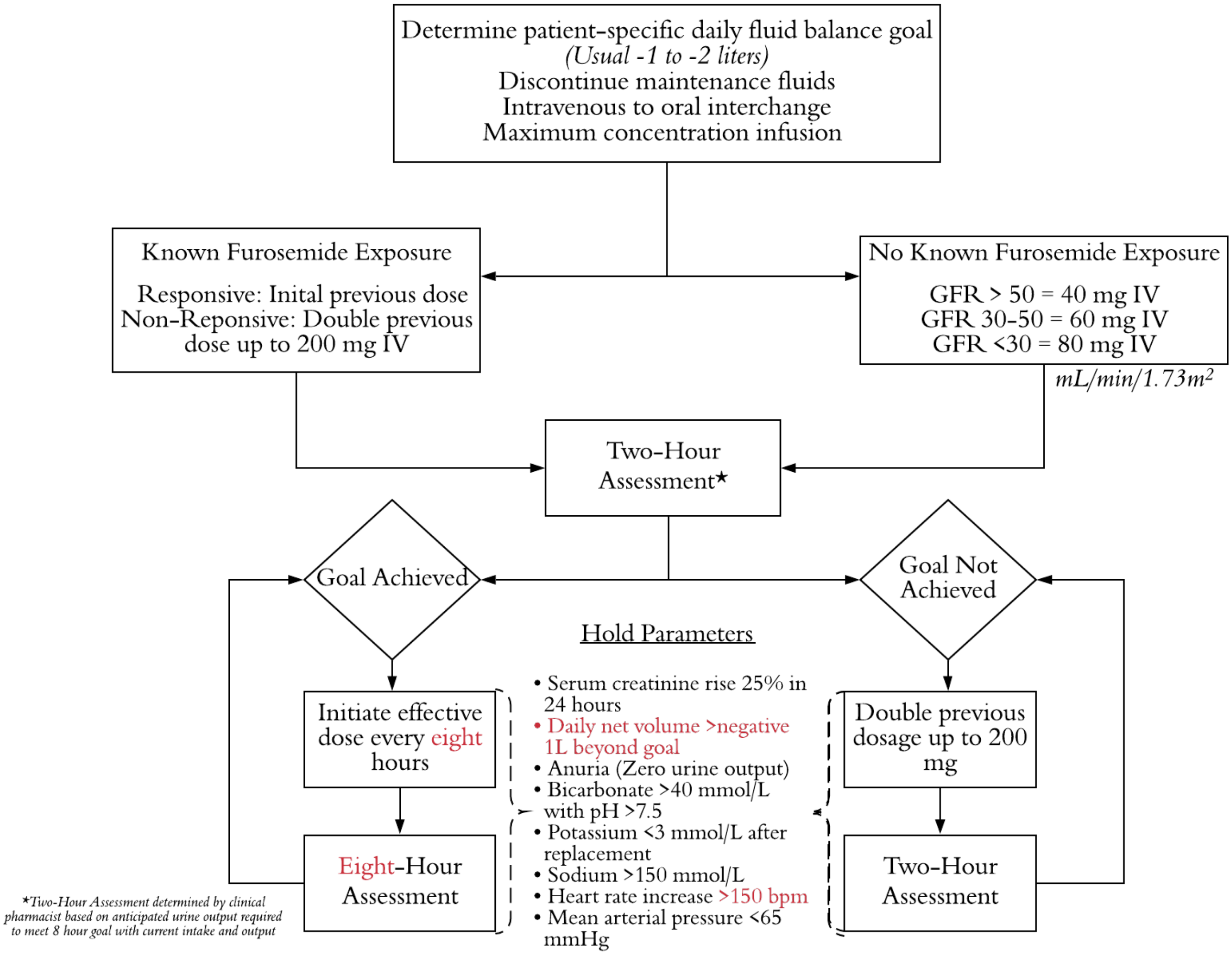

eTable 1. Study Inclusion and Exclusion Criteria

<table>
<tr><th>Inclusion Criteria</th><th>Exclusion Criteria</th></tr>
<tr><td rowspan="2">≥18 years old admitted to the pilot Medical Intensive Care Unit (MICU)</td><td>Comfort care decision to limit support or imminent death, as decided by MICU team</td></tr>
<tr><td>Nephrology consult for stage 3 acute kidney injury, defined as ≤0.3 mL/kg/hr urine output for at least 12 hours, serum creatinine (SCr) 3 times baseline, a SCr ≥4, or receipt of renal replacement therapy</td></tr>
<tr><td rowspan="2">Receiving mechanical ventilation with net positive or net even fluid balance or signs of fluid overload on chest x- ray or clinical examination</td><td>Acute treatment of any of the following:
1. Diabetic ketoacidosis/Hyperosmolar hyperglycemic state
2. Rhabdomyolysis with a creatine kinase level >5000 units/liter
3. Suspected hepatorenal syndrome</td></tr>
<tr><td>End-stage kidney disease or anuria (zero urine output for at least 12 hours)</td></tr>
<tr><td rowspan="3">Hemodynamically stable, defined as absence of vasopressor administration or bolus crystalloid administration within 12 hours, unless cardiogenic shock or norepinephrine <0.05 mcg/kg/min</td><td>Chronic restrictive, obstructive, neuromuscular, chest wall or pulmonary vascular disease resulting in severe exercise restriction, secondary polycythemia, severe pulmonary hypertension (mean PAP >40 mmHg), or respirator dependency preventing further mechanical ventilation wean</td></tr>
<tr><td>Pregnancy demonstrated via serum hCG</td></tr>
<tr><td>No history of neuromuscular disease that impairs ability to ventilate spontaneously, such as C5 or higher spinal cord injury, amyotrophic lateral sclerosis, Guillain-Barre Syndrome, and myasthenia gravis</td></tr>
</table>

eTable 2. Additional Baseline Characteristics

Parameter	Historical Cohort (n=273)	Intervention Cohort (n=91)	p-Value
**Past Medical History**			
Chronic Respiratory Failure[b]	89 (32.6)	28 (30.8)	0.746
DKA or HHS[c]	0 (0)	1 (1.1)	0.250
Hepatorenal Syndrome[c]	4 (1.5)	0	0.576
Pulmonary Hypertension[c]	0	0	1.000
Renal Transplant[c]	1 (0.4)	0	1.000
**Other Admission Parameters**			
Admission Weight (kilograms [kg])[a]	92 (74-110)	89 (68-117)	0.813
Rhabdomyolysis[c]	0 (0)	8 (8.8)	<0.001
Mean Arterial Pressure (mmHg)[a]	81 (73-91)	82 (71-91)	0.904
Heart Rate (bpm)[a]	91 (80-104)	88 (75-101)	0.178
Time Since Shock Resolution[a]	38 (10-72)	38 (6-77)	0.721
Home Furosemide[b]	31 (11.3)	6 (6.6)	0.193
Home Furosemide Dose (mg)[a]	40 (40-40)	40 (20-40)	0.294
**Laboratory Results**			
Serum Creatinine (mg/dL) [a]	0.86 (0.63-1.30)	0.80 (0.66-1.14)	0.378
Blood Urea Nitrogen (mg/dL) [a]	23 (15-34)	25 (15-37)	0.691
pH, Arterial[a]	7.38 (7.29-7.44)	7.42 (7.37-7.45)	0.009
pH, Venous[a]	7.37 (7.31-7.42)	7.43 (7.36-7.46)	0.104
Sodium (mmol/L) [d]	142 (5)	143 (4)	0.051
Potassium (mmol/L) [a]	4.1 (3.8-4.4)	4.1 (3.8-4.4)	0.382
Bicarbonate (mmol/L) [d]	27 (7)	29 (5)	0.121
Chloride (mmol/L) [d]	105 (6)	106 (6)	0.291
Albumin (g/dL)[a]	2.1 (1.7-2.5)	2 (1.5-2.2)	0.191

eTable 3. Primary Diagnosis Code Groups

	Historical Cohort (n=273)	Intervention Cohort (n=91)	p-Value
Cardiac Procedure or Disorder	9 (3.3)	2 (2.2)	0.738
Dermatologic Disorder	4 (1.5)	0 (0)	0.576
Gastrointestinal/Endocrine Disorder	9 (3.3)	2 (2.2)	0.738
Gynecology/Urology	2 (0.7)	3 (3.3)	0.102
Hematology/Malignancy	2 (0.7)	2 (2.2)	0.261
Immunologic Disorder	4 (1.5)	1 (1.1)	1.000
Liver Disease	8 (2.9)	2 (2.2)	1.000
Neurologic Disorder	9 (3.3)	7 (7.7)	0.076
Post-Operative	11(4.0)	3 (3.3)	1.000
Respiratory Failure	55 (20.1)	21 (23.1)	0.551
Sepsis or Infection	115 (42.1)	34 (37.4)	0.424
Toxicology	7 (2.6)	3 (3.3)	0.716

eTable 4. Subgroup Analysis of Pre- and Post-Modification Enrollment

	Coefficient	95% Confidence Interval	p-Value
Intervention Group	-3569.6	-5026—2113.0	<0.0001
Post Modification	1017.1	-718-2752.6	0.250
Overall p-value for model <0.0001<br>R^2 =0.0923			
Delta-Method Adjusted Predictions (n=364)			
	Margin	Standard Error	p-Value
Historical Group	474.7	246.5	0.055
Pre-Modification Intervention Group	-3094.9	697.4	<0.0001
Post-Modification Intervention Group	-2077.8	539.4	<0.0001

eTable 5. Subgroup Analysis for 72-Hour Post-Shock Fluid Balance Excluding Patients Included Based on Clinical Exam Criteria

	Number	Mean	95% Confidence Interval	Standard Deviation	p-Value
Pre-Intervention	57	-1300.4	(871.3-4382.2)	377.1	0.0038
Post-Intervention	27	-3927.1			

eTable 6. Interrupted Time Series Analysis

	Estimate	Approximate Standard Error	p-Value
Study Time	3.65	2.89	0.2078
Intervention Group	-2795.81	1005.2	0.0002
Slope Difference Between Cohorts	15.78	15.39	0.3058

eTable 7. Logistic Regression Model for All-Cause Mortality

	Odds Ratio	95% CI	p-Value
Ventilator Time to Furosemide	1.00	0.99-1.00	0.199
Net Volume Prior to Furosemide	1.00	0.99-1.00	0.861
SOFA	1.33	1.18-1.49	<0.001
Age	1.04	1.01-1.06	0.002
Intervention Group	0.24	0.09-0.68	0.007
Overall p-value for model <0.0001<br>Hosmer–Lemeshow goodness-of-fit test p = 0.139<br>C-statistic = 0.756			

eTable 8. Indication for Furosemide Dose Holds in Intervention Group

Indication	Count
Serum creatinine	8
Net Volume negative 1 liter beyond goal	20
Hypokalemia	1
Hypernatremia	3
Hypotension	8
Tachycardia	14
Unknown	13
Goal met/Protocol discontinuation	63
No holds	97

8 patients with holds for >1 safety reason

18 patients with hold for safety indication plus achievement of extubation or goal net volume status
